# Communicative signals support abstract rule learning by 7-month-old infants

**DOI:** 10.1038/srep25434

**Published:** 2016-05-06

**Authors:** Brock Ferguson, Casey Lew-Williams

**Affiliations:** 1 Department of Psychology, Northwestern University, 2029 Sheridan Rd., Evanston, IL, USA 60208; 2 Department of Psychology, Princeton University, Princeton, NJ, USA 08540.

## Abstract

The mechanisms underlying the discovery of abstract rules like those found in natural language may be evolutionarily tuned to speech, according to previous research. When infants hear speech sounds, they can learn rules that govern their combination, but when they hear non-speech sounds such as sine-wave tones, they fail to do so. Here we show that infants’ rule learning is not tied to speech *per se*, but is instead enhanced more broadly by communicative signals. In two experiments, infants succeeded in learning and generalizing rules from tones that were introduced as if they could be used to communicate. In two control experiments, infants failed to learn the very same rules when familiarized to tones outside of a communicative exchange. These results reveal that infants’ attention to social agents and communication catalyzes a fundamental achievement of human learning.

Much of what humans come to know about the world is learned from others in communicative contexts^1^,^2^,^3^,^4^. In language development, these contexts have been shown to promote word learning by engaging the cognitive mechanisms underlying infants’ learning about the sounds^5^,^6^,^7^ and meanings of words^8^,^9^,^10^,^11^,^12^,^13^. But learning about words and their meanings is only part of infants’ challenge in learning language. To become proficient speakers, infants must also learn about the abstract, grammatical rules that determine how words are combined into sentences^14^,^15^.

As a window into the origins of grammar learning, researchers have examined infants’ ability to learn and generalize abstract rules from input. In their seminal paper, Marcus and colleagues^16^ demonstrated that 7-month-old infants could detect a rule based on identity relations (e.g., ABB) in sequences of speech syllables (e.g., *le di di*) and generalize them to recognize new sequences as either consistent (e.g., *ko ga ga*) or inconsistent (e.g., *ko ga ko*, following ABA) with this rule. Other studies have replicated this finding, demonstrating that infants’ capacity for learning rules from speech is highly robust^17^,^18^,^19^, with evidence that even newborn infants detect rule-like regularities from speech^20^.

Infants’ rule learning from sounds other than speech is strikingly more limited. In one set of experiments, 7-month-olds failed to learn the same rules that they could learn successfully from speech when presented with sine-wave tones, animal sounds, or musical timbres^21^. These findings led to the proposal of a “speech advantage” in rule learning^22^, perhaps resulting from speech-specific adaptations that have evolved with our capacity for language^23^. Although evidence has since revealed that younger infants, at 4 months, can learn rules from non-speech sounds^24^, and that 7-month-olds can learn rules from visual stimuli^25^, a compelling alternative account of the gap between learning rules from speech and non-speech sounds at 7 months is still outstanding. What can account for the emerging speech advantage in infants’ rule learning?

In the present research, we propose and test one hypothesis; namely, that 7-month-olds’ rule learning is not engaged by speech *per se*, but rather by communicative signals in general. From birth, infants tune their attention to communicative stimuli including speech^26^,^27^, eyes^28^, and communicative gestures^29^. Furthermore, at 6 months (just prior to the age at which the speech advantage emerges^21^,^24^), infants’ behaviour and neurological patterns reveal an understanding that speech serves a communicative function^30^,^31^. We reasoned that the communicative status of speech, along with the attention infants devote to it, may have powerful consequences on infants’ learning. Just as communicative contexts enhance learning about words’ sounds and meanings, so too might they enhance the learning of abstract, grammar-like rules.

One means of testing this hypothesis is to compare infants’ learning from speech to their learning from other communicative signals. A prior study took this approach and asked whether 7-month-olds could learn rules from American Sign Language (ASL) gestures^22^. The researchers found that when rules were instantiated in gestures, infants’ learning was inconsistent across rule types (ABB versus AAB) – an inconsistency not seen when learning from speech – and thus concluded that communicative status alone is insufficient to promote rule learning. However, while signing gestures are a primary modality of communication for some infants, the participants in this study had not been previously exposed to ASL gestures. Thus their difficulties in rule learning could be explained by a failure to recognize these gestures as communicative actions. Their difficulties might also be explained by broader differences between learning rules from dynamic actions in the visual modality versus discrete sounds in the auditory modality.

We took a different approach to examining the hypothesis that communicative signals support infants’ rule learning. In four experiments, we assessed infants’ ability to learn rules from novel sounds (sine-wave tones) in communicative versus non-communicative contexts. In two experiments, infants’ experience with tones was designed to demonstrate that they served a communicative function; in two control experiments, the function of tones was ambiguous. We predicted that, if rule learning at this age was engaged by speech alone, infants should fail to learn regardless of whether or not the tones were communicative. In contrast, if infants’ attention and rule-learning abilities are engaged by communicative signals more broadly, then infants should learn rules more reliably when tones were introduced as a communicative signal than when their function was not clearly communicative.

## Experiment 1

In our first experiment, we adapted a previous experimental design in which infants demonstrated failure to learn rules from tones^21^. However, we made one crucial modification: prior to participating in the tone rule-learning task, we pre-exposed infants (*N* = 16, *M* = 7.56 months, 5 females) to a video in which two female actors had a brief conversation. In this video, one actor spoke in English, and the other actor responded using tones dubbed over her mouth movements. Prior work has demonstrated that this brief pre-exposure to tones in a communicative exchange can convince infants that tones are a communicative signal^13^. At issue in the present study was whether this newfound communicative status of tones would subsequently enhance infants’ ability to learn rules from tones. Infants were familiarized to four repetitions of 16 distinct tone sequences, each of which followed the same rule (e.g., an ABB rule consisting of the tone sequence *C F F*). Next, infants participated in 12 test trials in which their attention was drawn to a dynamic visual stimulus and they heard novel tone sequences following either the familiar rule (e.g., *G# A# A#*) or a novel rule (e.g., an ABA rule, as in *G# A# G#*).

We predicted that if infants could learn rules from tones after being exposed to tones as a communicative signal, they should look longer to the visual stimulus on novel test trials (which presented violations of the rule) than on familiar test trials. In contrast, if infants once again failed to learn rules from tones, they should look equally during both types of trials. To test these predictions, we compared infants’ mean looking times between familiar and novel trials using a paired *t*-test to assess whether they were significantly different from chance.

**Figure 1:**
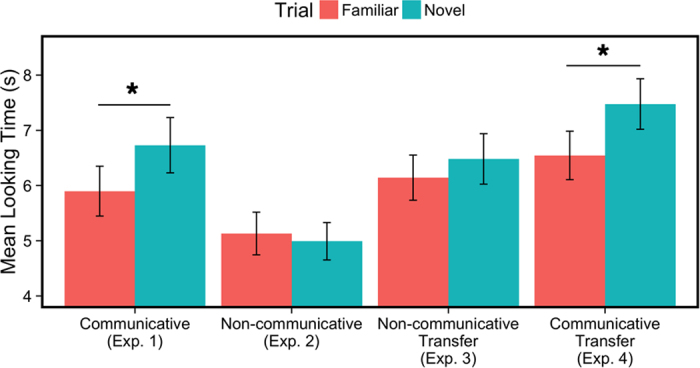
Infants’ average attention to Novel and Familiar trials during the test phases of Experiments 1–4. In Experiments 1 and 4, which pre-exposed infants to tones as a communicative signal, infants showed a reliable preference for novel trials over familiar trials. In Experiment 2, which pre-exposed infants to tones in a non-communicative video, and in Experiment 3, which did not pre-expose infants to tones, infants looked for approximately equal lengths of time on both trial types. Error bars represent +/− 1 SEM (between subjects).

Our results (see Fig. 1) revealed that the communicative pre-exposure video had a clear influence on learning. Infants reliably learned rules from tones, attending longer to novel trials (*M*_*diff*_ = 0.83s, *SD* = 0.81) than familiar trials during the test phase, *t*(15) = 4.12, *p* < 0.001, *d* = 1.03. This difference in looking times at test indicates that infants succeeded in learning rules from tones after only a brief exposure to tones as a communicative signal, thereby revealing the power of communicative status in general (and not speech alone) in engaging their rule learning.

## Experiment 2

In a second experiment, we examined two alternative explanations of infants’ performance in Experiment 1. One possibility is that the pre-exposure video merely made the tones more familiar and, in turn, made them easier for infants to process. A second possibility is that the social nature of the pre-exposure video increased infants’ interest in the task and, in turn, their learning. To test whether stimulus familiarity and social engagement alone are sufficient for infants to learn rules from tones, we modified our design to include a new pre-exposure video that held these factors intact without introducing the tones as communicative. In this new video, infants (*N* = 16, *M* = 7.46 months, 6 females) observed the same two actors socially cooperating in a simple task. The video soundtrack consisted of the same speech and tone sounds from Experimenter 1’s communicative pre-exposure video, now occurring without an identifiable source. If the communicative status of the tones was critical in enhancing infants’ learning in Experiment 1, we predicted that infants would fail to learn rules from tones after viewing this social but non-communicative video. However, if mere exposure to tones in a social context was sufficient, then infants should succeed in learning tones after viewing this vignette.

This prediction was borne out in our results: Infants’ attention during test did not differ (*M* = −0.14s, *SD* = 1.29) between novel and familiar test trials, *t*(15) = −0.44, *p* = 0.67, *d* = −0.11. A 2 (Experiment) × 2 (Trial Type) ANOVA confirmed that infants’ learning between the two experiments was different, revealing a main effect of Experiment (*F*(1,30) = 4.86, *p* = 0.035) and an interaction between Experiment and Trial Type (*F*(1,30) = 6.56, *p* = 0.016). Thus neither familiarity with tones nor general social engagement could entirely account for the influence of the communicative pre-exposure video on infants’ learning in Experiment 1.

Collectively, the first two experiments provide evidence that communicative relevance enhances infants’ rule learning, but we wanted to evaluate this hypothesis in the context of a stronger test of rule generalization, from one kind of sound (tones) to another (speech). Although infants often struggle to generalize knowledge from one stimulus to another^32^, our interest in this question was driven by a compelling demonstration of transfer in infants’ rule learning from speech reported by Marcus *et al.*^21^. In their experiments, 7-month-old infants were familiarized to rules in speech sounds and then tested on various non-speech sounds, including tones, animal sounds, and musical timbres. Succeeding in this task not only required infants to learn the rule but, later, to transfer this rule to a novel sound. They found that infants could in fact learn rules from speech and transfer them to non-speech sounds, documenting that the rules infants learned from speech were robust and highly generalizable. In Experiments 3 and 4, we were interested in the conditions under which infants could transfer rules in the opposite direction, from tones to speech.

## Experiment 3

To begin addressing this issue, in our third experiment, we familiarized infants (*N* = 16, *M* = 7.48 months, 4 females) to rules in tones and then tested them on speech sounds, thus reversing the design of Marcus *et al.*^21^. This experiment served as our first look at infants’ ability to transfer rules from tones to speech, and it did not include a pre-exposure phase. In Experiment 2 and prior research, 7-month-olds have failed to learn rules from tones without a pre-exposure to tones as a communicative signal^21^,^24^. We therefore expected that infants would fail to discriminate the test sequences in the tones-to-speech generalization task in Experiment 3. Nevertheless, this was an important control experiment because the mere presence of speech – even during the test phase alone – might enable infants to successfully discriminate different rules. Our results confirmed that infants’ rule learning from tones (even when tested on speech) is not reliable: when familiarized to tones and tested with speech, infants once again did not show a preference at test (*M* = 0.34s, *SD* = 1.41), *t*(15) = 0.96, *p* = 0.35, *d* = 0.24. The results of Experiment 3 indicated that infants could not transfer rules from tones to speech, but in tandem with Experiments 1 and 2, it opened the possibility that infants may succeed in doing so if introduced to tones in a communicative context.

## Experiment 4

In a fourth experiment, we adapted the tones-to-speech transfer task to include the communicative pre-exposure video from Experiment 1. Would infants succeed in learning rules from tones – and transferring them to discriminate sequences of speech – after observing tones as an engaging, communicatively relevant signal? Once again, we found that this brief pre-exposure video impacted infants’ (*N* = 16, *M* = 7.43 months, 5 females) rule learning. Infants looked reliably longer during novel trials than during familiar trials at test (*M* = 0.93, *SD* = 1.63), *t*(15) = 2.29, *p* = 0.036, *d* = 0.57. This experiment further supports the power of communication in helping infants learn abstract rules from tones, but also goes further to reveal that communicative status supports the transfer of rules from tones to a different auditory signal.

## Discussion

These experiments collectively document a previously unexplored influence of communicative contexts on the origins of learning in infancy; namely, that communicative signals (even those to which the infant was just introduced) support the learning of abstract rules. We manipulated the communicative status of tones by embedding them into a natural conversation between two women, and in doing so, altered infants’ ability to learn and generalize the underlying structure of tone sequences.

The four experiments provide a new explanation for why 7-month-olds reliably learn rules from speech but not from non-speech sounds: speech may engage infants’ rule learning by its status as a communicative signal. This explanation is consistent with findings that the speech advantage emerges between 4 and 7 months, the time period in which we see the first evidence that infants treat speech as a communicative signal^24^,^30^,^31^. Although 4-month-olds may learn rules from many kinds of stimuli (including tones in non-communicative contexts), 7-month-olds may learn rules from some stimuli (e.g., tones in communicative contexts) but not others (e.g., tones in non-communicative contexts) because they have come to privilege stimuli that are most relevant in their environment, such as the communicative signals of conspecifics (mirroring ‘perceptual narrowing’ processes in face and speech perception^33^,^34^,^35^). Evidence that infants preferentially attend to communicative signals such as speech^26^,^27^ offers a potential mechanism for this influence: relative to ambiguous sounds, communicative signals may differentially engage infants’ attention either in quantity or in kind, thus enhancing their ability to detect underlying structure. Similar effects of attention effects have been documented in adults in other forms of pattern learning, such as statistical learning^36^,^37^,^38^. If this attentional account is correct, then other stimuli that engage infants’ attention, such as cross-modally congruent stimuli^39^,^40^ and animate entities^41^,^42^, might also enhance rule learning (see, for example, recent work on cross-modal rule learning with infants^43^,^44^).

An open question concerning the role of communicative signals in particular is which elements of the communicative exchange presented to infants in our experiments were critical to engaging their learning. Our brief, naturalistic pre-exposure videos included a host of social and perceptual cues that might engage infants’ attention and suggest to them that tones served a communicative function, any one of which might be necessary or sufficient for enhancing later learning. For example, it could be the tones’ participation in a contingent, turn-taking-like exchange^4^,^45^, their co-occurrence with speech, or their production by a human agent^46^,^47^ that led infants to recognize them as communicative. At another perceptual level, the auditory-visual synchrony between the tones and the actors’ dynamic facial movements^40^ and actions^39^,^48^ may have underpinned the observed learning effect. Further research manipulating details of infants’ experience with novel sounds will be critical for understanding the sources of infants’ attentional engagement and interpretation of communicative signals.

The present findings reveal a mechanism by which social cognition – specifically, perception of communicative information – shapes a fundamental process underlying infants’ learning. We demonstrated that communicative contexts enhance learning and generalization of structured input, much like the rules that constitute grammar. Even if the observed effects were fleeting, communicatively relevant signals in the lives of infants are not. Indeed, much of what they must learn about the world is introduced in communicative contexts. Therefore, the effect of communicative signals on infants’ learning on a moment-to-moment basis may have profound consequences on their learning across the protracted scale of development. Infants’ sensitivity to communicative contexts may contribute not only to explaining the longstanding mystery of how human infants learn language so quickly, but also why children who are deprived of such contexts^49^ or lack the capacities with which to engage them^50^ may be hindered in the same pursuit.

## Method

### Participants

Our final analyses included a total of 64 English-learning infants ranging from 7.0 – 7.99 months of age. English accounted for 75% or more of each participant’s language input from caregivers. An additional 36 participants were excluded for technical errors (*N* = 4), failing to contribute at least 8 test trials (*N* = 20), looking (on average) greater (*N* = 2) or less (*N* = 2) than 2.5 *SD* away from the experiment mean, hitting the trial ceiling (16 sec; see below) on 8 or more test trials (*N* = 3), or excessive irritability during the familiarization (*N* = 1) or test (*N* = 4) phases. Exclusion rates did not vary by experiment (*χ*(3) = 5.11, *p* = 0.16).

Experiments were performed with approval and under the accordance of the relevant guidelines established by the Institutional Review Board at Northwestern University. We obtained informed consent from infants’ caregivers at the beginning of each lab visit.

### Materials and Procedure

Each experiment followed the same structure and included a pre-exposure (aside from experiment 3), familiarization, and test phase. Custom MATLAB software (R2010b, Mathworks Inc.) was used to control each phase. In the pre-exposure phase (1 min), infants observed one of two brief videos that included sine-wave tones (adapted from those in Ferguson & Waxman^13^). These tones ranged from notes *C3-G3*, overlapping with those during the familiarization phase, but did not include distinct ABB, AAB, or ABA triplets that might have pre-familiarized infants to the rule. In experiments 1 and 4, this video portrayed the tones as a communicative signal: one person in the video produced the tones (dubbed over her mouth movements) in conversation with another person, who responded in English. In experiment 2, this video included the same two people collaborating on a task (mixing liquids with spoons) while the audio track from the conversational video played in the background, uncoupled from the actors’ movements.

After pre-exposure, infants continued immediately to the familiarization phase (2 min, 34 sec) in which they heard an audio track of 16 distinct tone sequences arranged to form a rule (either ABB or ABA, randomly assigned between-subjects). The 16 sequences in each condition were randomly arranged into 37-sec blocks; infants heard the block corresponding to their condition repeated 4 times. Tone sequences were constructed using notes *C, C#, D, Eb, E, F, F#*, and *G*. The intervals between the two notes in a sequence ranged from 1 (e.g., *C# C# D*) to 5 semitones (e.g., *C C F*). Each tone was 300 msec in duration with 250 msec between tones, and sequences were separated by 1000 msec of silence. Tone sequences played for the entire duration of the familiarization phase. While infants heard the tones, they were also trained to look left and right in a head-turn preference procedure consisting of three monitors positioned to the center, left, and right of the infant. Throughout the familiarization phase, infants were repeatedly oriented to the center monitor using a dynamic visual stimulus (a picture of a looming face). When they looked at the monitor, the visual stimulus disappeared and appeared on one the monitors on the infant’s right or left. If the infant oriented toward the side monitor, the image remained on the monitor for as long as the infant fixated it. After they looked away from the monitor, or if they failed to look to the monitor at all for 60 sec, the image disappeared and then re-appeared on the center monitor, at which point this cycle began again. The audio stimuli used in the familiarization phase played continuously and were not time-locked to the visual stimuli. This approach is commonly used to familiarize infants in the head-turn preference procedure and has been used successfully in prior infant studies^51^,^52^.

After the familiarization phase, infants continued immediately to the test phase consisting of 12 test trials organized into 3 blocks. The total duration of the test phase was in part controlled by the infant. In each block, infants heard two familiar test trials and two novel test trials in random order. On familiar trials, infants heard two sequences consisting of novel sounds arranged to match the rule heard during familiarization (either ABB, ABA, or AAB); on novel trials, infants heard two sequences consisting of the same sounds, but arranged to match a different rule than that heard during familiarization. In Experiments 1 and 2, the test sounds consisted of notes *G#, A, Bb*, and *B*, arranged into sequences with whole tone intervals, e.g., *G# Bb Bb* (for an ABB rule) and *B A B* (for an ABA rule). In Experiments 3 and 4, test sounds consisted of synthesized speech syllables *po, ba, ko*, and *ga* (e.g., *ba po po*), selected to match those used in prior studies in which 7-month-olds learned rules from speech^16^. Infants heard the same two novel sequences in alternation in each novel trial and the same two familiar sequences in alternation in each familiar trial; however, the order of the sequences was randomized across trials. Infants’ attention during each trial was measured in the head-turn preference procedure. Each trial began by orienting the infant’s attention to the center monitor. Once the infant looked to the center, the visual stimulus disappeared and re-appeared on the left or right. After the infant looked to the side monitor, the two sequences for that trial began to play in alternation; this continued until the infant looked away for 2 consecutive seconds or until infants looked for the maximum length of 16 s. If the infant failed to orient to the side monitor, or if they looked for less than the required 2 s, the trial was skipped and was attempted again at the end of the test phase.

### Coding

A trained coder observed each infant using a hidden camera beneath the center monitor and indicated when the infant was looking to the center, left, and right monitors by pressing buttons. Due to the nature of our experimental manipulation and the design of the testing room, experimenters were able to hear the pre-exposure video and infer the infant’s experimental condition while coding their looking behaviors. To ensure that coding was unbiased, a separate set of trained coders – blind to experimental condition and our hypotheses – re-coded each video offline, frame-by-frame, in silence (except for 5 sessions which failed to be recorded; *N* = 4 in Exp. 3, *N* = 1 in Exp. 4). We performed several analyses comparing the offline and online codes to assess the reliability of online coding and detect whether there was any bias in the lengths of test trials. First, we assessed the correlation between the looking times of trials coded offline and online, and found them to be highly correlated (*r* = 0.98). This suggests that the online coding was generally accurate, and is consistent with other head-turn preference studies^53^,^54^. Second, we examined trial errors that might have affected looking time values. We considered an online coder to have committed an error in a trial if (1) the trial did not end after a look-away of 2.5 sec or longer (allowing a 500 msec response time buffer), (2) the trial ended with a look-away of less than 1.5 sec (again allowing a 500 msec buffer), or (3) the trial ended during a look. In the head-turn preference paradigm, some errors are inevitable because the online coders’ response times are delayed by their perceptual judgments (determining whether the infant is looking) and motor planning (executing a movement to release or press a button). Critically, however, we found online coding errors to occur infrequently, with 2.96% of trials erroneously lengthened and 4.06% of trials erroneously shortened. A mixed-effects regression (in R’s *lme4* syntax: MeanRate ~ ErrorType*TrialType + (1 + ErrorType + TrialType | Subject)) within communicative (Experiments 1 and 4) and non-communicative conditions (Experiments 2 and 3), respectively, did not reveal a statistically reliable interaction between Error Type and Trial Type in either group (communicative: *p* = 0.86, non-communicative: *p* = 0.14). These results indicate that the experimenter’s tendency to erroneously lengthen or shorten trials did not reliably differ between conditions in which infants were predicted to successfully versus unsuccessfully learn abstract rules. Finally, we re-analyzed each experiment after removing trials that contained one or more errors, and found very similar effects to those reported in the main text: Infants in Experiment 1 showed a preference for novel vs. familiar test sequences (*M* = 0.73 sec, *SD* = 1.09, *d* = 0.67, *t*(15) = 2.68, p = 0.017), as did infants in Experiment 4 (*M* = 1.01 sec, *SD* = 1.85, *d* = 0.55, *t*(14) = 2.12, *p* = 0.053); in contrast, infants in Experiment 2 (*M* = −.40, *SD* = 1.35, *d* = −.30, *t*(15) = −.43, *p* = 0.25) and Experiment 3 (*M* = 0.73 sec, *SD* = 1.86, *d* = 0.39, *t*(11) = 1.22, *p* = 0.20) did not show strong preferences in either direction.

## Additional Information

**Data availability**: The data, analyses, and code in the paper are archived at https://github.com/brockf/ datashare/tree/master/COMMRULE.

**How to cite this article**: Ferguson, B. and Lew-Williams, C. Communicative signals support abstract rule learning by 7-month-old infants. *Sci. Rep.* **6**, 25434; doi: 10.1038/srep25434 (2016).

## Supplementary Material

Supplementary Information
