# Supplementary material for: Communicative signals support abstract rule learning by 7-month-old infants
Source: Sci Rep. 2016 May 6;6:25434. doi: 10.1038/srep25434 (PMC4858667; doi:10.1038/srep25434)
Supplement: Supplementary Information [file srep25434-s1.pdf]

Communicative signals support abstract rule learning by 7-month-old infants:

Supplementary Information

Brock Ferguson[1] and Casey Lew-Williams[2]

Affiliations:

[1] Northwestern University

[2] Princeton University

Supplementary Information

Additional analyses: Two aspects of Experiments 3 and 4 motivated us to perform confirmatory analyses using hierarchical linear models of infants' looking at test on a trial-by-trial scale. First, because the transfer experiments introduced a new sound during the test phase, we observed an increase in their attention at the beginning of the test phase followed by a significant decline in attention for the duration of the test, a pattern that we did not observe in the first two experiments. A model predicting infants' looking time during each test trial confirmed this by revealing a significant interaction between trial number and experiment ($\chi(6)=14.85$, $p=.021$). Follow-up models indicated a significant decline in attention during the test phase in experiments three and four ($\chi(1)=15.26$, $p<.001$), but not in experiments one and two ($\chi(1)=3.18$, $p=.075$). Second, in experiments three and four, a relatively high number of infants exhibited excessive fussiness shortly after beginning the test phase and failed to complete enough test trials. By modeling infants' data in these experiments trial-by-trial, we could control for the randomized order of presentation of novel and familiar trials and, furthermore, include partial data from infants who were excluded for failing to contribute the required number of test trials.

These models confirmed the pattern of results reported in the main text. In experiment three, in which infants did not have any pre-exposure to tones, there was a main effect of Trial Number ($\beta=-.26$, $p=.003$) but no effect of Trial Type (all other p's $> .25$). In contrast, in experiment four, in which infants were pre-exposed to tones as a communicative signal, there were main effects of both Trial Number ($\beta=-.33$, $p=.011$) and Trial Type ($\beta=.80$, $p=.044$). This main effect of Trial Type remained significant even when including partial data from all infants who had been excluded for failing to complete the study ($N=27$, $\beta=.93$, $p=.048$), thus confirming

that infants' overall preferences at test were not distorted by the randomization of trials or infant attrition.
